# Evaluation of spexin levels in euthyroid patients with Hashimoto thyroiditis and its relation to autoimmunity

**DOI:** 10.1097/MD.0000000000040321

**Published:** 2024-10-25

**Authors:** Oguzhan Aksu, Ummugulsum Can, Selma Ozlem Celikdelen, Betul Cigdem Yortanli, Muhammet Cemal Kizilarslanoglu, Ayse Gunay

**Affiliations:** a Department of Internal Medicine, Division of Endocrinology and Metabolism, University of Health Sciences, Konya, Turkey; b Department of Biochemistry, Konya State Hospital, Konya, Turkey; c Department of Internal Medicine, Konya State Hospital, Konya, Turkey; d Department of Internal Medicine, Division of Geriatric Medicine, University of Health Sciences, Konya, Turkey; e Department of Clinical Pharmacy, Konya State Hospital, Konya, Turkey.

**Keywords:** Autoimmune processes, Hashimoto thyroiditis, Spexin

## Abstract

Hashimoto thyroiditis (HT) is chronic lymphocytic thyroiditis. Cytokines and chemokines such as tumor necrosis factor-alpha, interferon-gamma, and interleukin-1 beta originating from immune cells are involved in the etiopathogenesis of HT. Spexin (SPX) is a recently identified novel peptide hormone consisting of 14 amino acids and has been demonstrated in follicle epithelial cells in thyroid tissue. SPX has been shown to affect the inflammatory response and play a role in its regulation in various diseases. There is a need for markers for diagnosis and treatment of HT patients with negative antibody levels. We found that there is no study in the literature that investigates the HT and the role of spexin in this inflammatory process. Forty-five patients aged 18 to 70 years with HT or newly diagnosed HT and 42 healthy subjects as the control group were included in the study. Patients in the HT group were divided into 3 categories according to ultrasound findings. Mild heterogeneity was called grade 1 (G1), moderate heterogeneity was called grade 2 (G2), and high heterogeneity was called grade 3 (G3). Laboratory parameters and anthropometric measurements of all patients participating in the study were performed, and SPX was measured by the ELISA method. There was no significant difference between the HT and control groups in terms of SPX levels (*P* = .27). In HT subgroup analysis, SPX levels were found to be borderline statistically significantly higher in the G2 group, where antibody levels were higher compared to other groups (*P* = .061). In our study, we evaluated SPX levels in HT patients, which has never been done before in the literature. We found high SPX levels in HT patients with high antibody levels. Multicenter studies with high case series, especially at the tissue level, are needed to fully explain the role of SPX in HT immunoetiopathogenesis and to understand immune-checkpoint pathways more clearly.

## 1. Introduction

Hashimoto thyroiditis (HT) is a common autoimmune disease, especially affecting women, with a 10 to 17% incidence.^[1,2]^ It is characterized by chronic lymphocytic inflammation and the destruction of the thyroid gland. Antibodies against thyroid peroxidase enzyme (TPO) and thyroglobulin (TG) are responsible for the destruction, and due to this process, hypothyroidism develops in at least 20% to 30% of patients.^[3]^ Genetic factors play a role in 70% to 80% of the cases. In 20% to 30% of the patients, environmental factors were found to be the root cause.^[2]^

Cytokines are secretory proteins that mediate immune and inflammatory reactions and play important roles in the initiation and maintenance of thyroid autoimmunity.^[4]^ Cytokines and chemokines such as tumor necrosis factor-α (TNF-α), interferon-gamma, and interleukin (IL) IL-1β, IL-6, IL-12, IL-13, and IL-18 originating from infiltrating and activated immune cells are involved in the etiopathogenesis of HT.^[5]^ SPX, or neuropeptide Q is a peptide that was first identified in the rat stomach and esophagus in 2007 using bioinformatic techniques.^[6]^ SPX is released from many central and peripheral tissues. SPX mRNA and SPX cytoplasmic immunoreactivity have been found in the skin, lung, stomach, small intestine, colon, liver, pancreatic islets, thyroid, adrenal gland, visceral fat, and kidney.^[7]^

Circulating SPX levels have been found to be lower in obese people, patients with type-2 diabetes mellitus (DM), and insulin resistance (IR).^[8]^ Recent studies have shown that SPX affects the inflammatory response in various diseases and is involved in illness regulation.^[9]^

Based on the fact that SPX is synthesized from thyroid tissue and plays a role in the inflammatory process, we found that there is no study in the literature on HT that develops due to a destructive damage that occurs after inflammatory and autoimmune processes, and we aimed to evaluate the role of SPX in this inflammatory process. There is a need for markers for diagnosis and treatment of HT patients with negative antibody levels.

## 2. Materials and methods

We conducted a single-center prospective study. Forty-five patients aged 18 to 70 years with a known or newly diagnosed with HT who were admitted to the endocrinology outpatient clinic and 42 healthy subjects as the control group that enrolled randomly were included in the study. Other autoimmune/systemic diseases besides thyroid disease and other metabolic diseases that can cause inflammation excluded in the study. For the diagnosis of HT, serum TSH, free T3, free T4 (FT4), anti-TG, and anti-TPO levels were measured in all patients including control subjects, and thyroid ultrasound was performed by a specialist with 15 years of experience. For the diagnosis of HT, serum TSH, free T3, FT4, anti-TG, and anti-TPO levels were measured in all patients, and thyroid ultrasound was performed by a specialist with 15 years of experience. None of the patients were on a medication that can alter the immune system. Patients in the HT group were divided into 3 groups according to ultrasound findings. The group with mild enlargement of the thyroid gland and echogenicity resembling normal thyroid tissue was called mildly heterogeneous, G1, the group with ultrasound findings involving <1/3 of the thyroid gland, including multiple hypoechoic foci, was called moderately heterogeneous, G2, and the group with much more hypoechoic than thyroid tissue and even the echo of the anterior neck muscles was called highly heterogeneous, G3. Ultrasonography of the patients in the control group was also performed and confirmed to be normal. Healthy individuals without any known disease were included as the control group. Patients with obesity (body mass index, BMI > 30 kg/m^2^ according to the World Health Organization insulin resistance) impaired fasting glucose and diabetes mellitus, patients with another autoimmune thyroid disease, and patients with any sign of infection at the time of the examinations were excluded.^[10]^

Anthropometric measurements such as height, weight, BMI, fasting blood glucose, insulin level, insulin resistance (HOMA-IR), and Hb1Ac levels were measured to identify obesity, prediabetes, and type-2 DM. SPX was measured by the ELISA method in all patients participating in the study. Blood samples were centrifuged and stored at −80 °C until analysis. After the measurements, the patients were divided into 3 groups (tertiles) according yo SPX levels.

### 2.1. Statistical analysis

Statistical analyses were performed in the SPSS (Statistical Package for Social Science) 22.0 computer program. Frequency (n), percentage (%), mean ± standard error, min (minimum)–max (maximum), Q25–Q75 (median values and 1st and 3rd quartile values) were used to evaluate the data obtained from the study. The normality of the data was checked by the Kolmogorov–Smirnov normality test. For statistical significance, the Chi-square (χ^2^) test was used for the comparison of categorical data, the *T* test was used for the comparison of 2 normally distributed groups for the comparison of continuous variables, and the Mann–Whitney *U* test, a non-parametric test, was used for the comparison of non-normally distributed groups. Pearson and/or Spearman correlation analysis was performed for the significance level between continuous data. All analyses were performed at a 95% confidence interval. For statistical significance, the *P* < .05 level was accepted as significant.

## 3. Results

A total of 87 patients, 45 with HT diagnosis and 42 healthy patients, were included in our study. The demographic, clinical, and laboratory values of all participants are shown in Table 1. The majority of the patients were female (79.3%). According to ultrasound examination, 33.3% (n = 15) of HT patients had G1, 46.7% (n = 21) had G2, and 20% (n = 9) had G3. The mean age was significantly higher in the HT group compared to the control group, 39 (18–67) and 29 (21–50), respectively (*P* < .001). BMI was significantly higher in the HT group compared to the control group (27 ± 4 and 24 ± 4, *P* = .011, respectively). FBG, HbA1c, insulin, and HOMA-IR were not significantly different in both groups. The FT4 level was significantly higher in the HT group 14 (9–18) compared to 12 (9–15) in the control group (*P* = .007). When both groups were compared, TSH levels were significantly higher in patient groups 2.5 (0.01–12.00) and 1.75 (0.50–3.43), respectively (*P* = .044).

**Table 1:** Comparison of the continuous and categorical variables between control and disease groups.

Parameters	Total n = 87	Control group n = 42	Hashimoto disease n = 45	*P* value
Gender (female)	69 (79.3)	30 (71.4)	39 (86.7)	.080
Co-morbidities, n (%)	0 (0.0)	0 (0.0)	0 (0.0)	–
Thyroid ultrasound findings, n (%)				
Mild heterogeneity	15 (17.2)	0 (0.0)	15 (33.3)	**<.001**
Moderate heterogeneity	21 (24.1)	0 (0.0)	21 (46.7)	
High heterogeneity	9 (10.3)	0 (0.0)	9 (20.0)	
Normal findings	42 (48.3)	42 (100.0)	0 (0.0)	
Age (years)	32 (18–67)	29 (21–50)	39 (18–67)	**<.001**
Height (cm)	165 ± 8	167 ± 8	164 ± 9	.161
Weight (kg)	70 ± 13	68 ± 13	72 ± 13	.199
BMI (kg/m^2^)	26 ± 4	24 ± 4	27 ± 4	**.011**
FBG (mg/dL)	85 (72–105)	84 (75–100)	86 (72–105)	.135
HbA1c (%)	5.3 (4.7–6.2)	5.3 (4.7–6.0)	5.4 (4.9–6.2)	.220
Insulin (mU/L)	8.2 ± 2.8	8.2 ± 2.8	8.1 ± 2.8	.820
fT3 (ng/L)	3.1 ± 0.4	3.1 ± 0.4	3.1 ± 0.5	.934
fT4 (ng/L)	12 (9–18)	12 (9–15)	14 (9–18)	**.007**
TSH (mU/L)	1.89 (0.01–12.00)	1.75 (0.50–3.43)	2.5 (0.01–12.00)	**.044**
Anti-TG (U/mL)	158 (12–1089)	–	158 (12–1089)	–
Anti-TPO (U/mL)	239 (9–600)	–	239 (9–600)	–
HOMA-IR	1.73 (0.44–3.82)	1.67 (0.44–2.93)	1.78 (0.54–3.82)	0.946
Spexin level (pg/mL)	45.8 (42.2–405.4)	45.1 (42.2–204.1)	46.6 (42.2–405.4)	0.275

The bold values in the table indicate statistical significance.

Anti-TG = anti-thyroglobulin, anti-TPO = anti-thyroid peroxidase, BMI = body mass index, FBG = fasting blood glucose, HbA1c = hemoglobin A1c, HOMA-IR = HOMA insulin resistance score, TSH = thyroid stimulating hormone.

**Figure 1.**
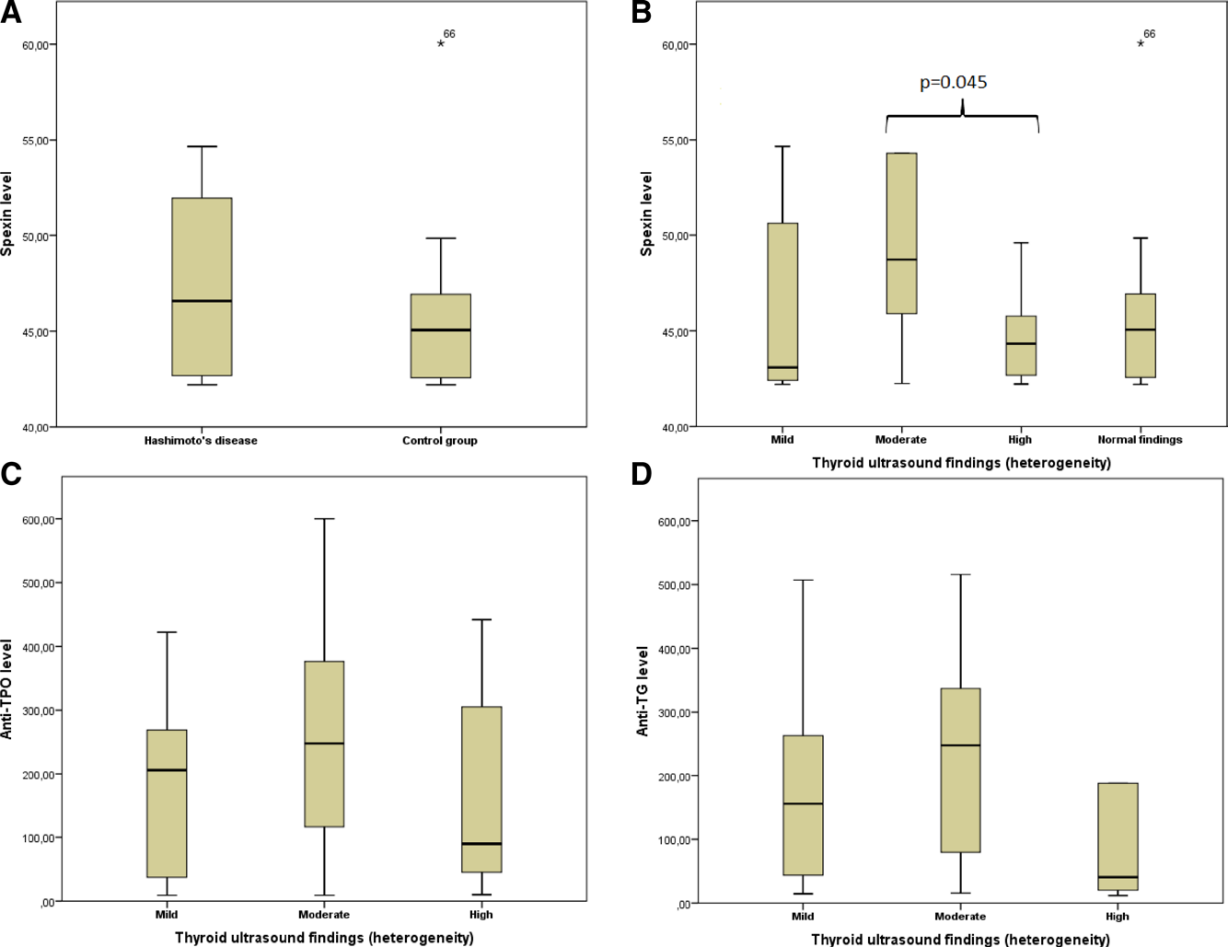
Serum spexin and autoantibody levels. (A) Serum spexin levels were similar between control and disease groups. (B) Serum spexin levels were compared between four groups according to the thyroid ultrasound findings and found to be significantly higher in the moderate group than in the high group (*P* = 0.045). (C and D) Anti-TPO and anti-TG levels were similar between groups categorized according to the thyroid ultrasound findings in the disease group. TG = thyroglobulin, TPO = thyroid peroxidase enzyme.

There was no significant difference between the HT and control groups in terms of SPX levels (46.6 (42.2–405.4) and 45.1 (42.2–204.1), *P* = .27, respectively). In HT subgroup analyses, the mean age was significantly higher in the G2 group than in the G1 and G3 groups (45 (23–66), 32 (18–67), and 36 (23–54), respectively (*P* = .038, Table 2)). The mean body weight was significantly higher in the G2 group than in the G1 group (77 ± 11 and 63 ± 10, respectively, *P* = .002). Similarly, BMI was significantly higher in the G2 group than in the G1 group (28 ± 4, 24 ± 42, respectively, *P* = .004). In the HT subgroup, SPX levels were significantly higher in the G2 group compared to G1 and G3 groups: 48.7 (42.2–405.4), 43.1 (42.2–84.7), and 44.3 (42.2–88.7), *P* = .061 (Table 2, Fig. 1).Correlation analysis between SPX level and other numerical parameters showed no statistically significant difference (*P* > .05 for all parameters). HT rate was significantly higher in group 3 compared to the group 2 and SPX levels were higher in tertile 3 (Table 3).

**Table 2:** Comparison of the study’s variables according to the thyroid ultrasound findings in the disease group.

Parameters	Mild heterogeneity n = 15	Moderate heterogeneity n = 21	High heterogeneity n = 9	*P*-value
Gender (female)	15 (100.0)	17 (81.0)	7 (77.8)	.172
Co-morbidities, n (%)	0 (0.0)	0 (0.0)	0 (0.0)	–
Age (years)	32 (18–67)	45 (23–66)	36 (23–54)	**.038**
Height (cm)	162 ± 6	164 ± 10	168 ± 10	.324
Weight (kg)	63 ± 10^1^	77 ± 11*	74 ± 14	**.002**
BMI (kg/m^2^)	24 ± 4†	28 ± 4†	27 ± 4	**.004**
FBG (mg/dL)	86 (77–100)	90 (78–105)	84 (72–99)	.103
HbA1c (%)	5.2 (4.9–6.1)	5.6 (4.9–6.1)	5.3 (5.1–6.2)	.318
Insulin (mU/L)	7.4 ± 2.2	8.3 ± 3.0	8.8 ± 3.0	.453
fT3 (ng/L)	3.1 ± 0.6	3.2 ± 0.4	3.0 ± 0.3	.702
fT4 (ng/L)	12.5 (9.5–18.0)	13.7 (9.0–17.0)	14.8 (9.6–17.9)	.319
TSH (mU/L)	2.7 (0.1–12.0)	2.8 (0.01–8.58)	1.2 (0.29–4.66)	.243
Anti-TG (U/mL)	156 (14–507)	248 (15–1089)	41 (12–1089)	.329
Anti-TPO (U/mL)	206 (9–422)	248 (9–600)	90 (10–442)	.553
HOMA-IR	1.6 ± 0.5	1.9 ± 0.7	1.9 ± 0.7	.452
Spexin level (pg/mL)	43.1 (42.2–84.7)	48.7 (42.2–405.4)‡	44.3 (42.2–88.7)‡	**.061**

The bold values in the table indicate statistical significance.

Anti-TG = anti-thyroglobulin, anti-TPO = anti-thyroid peroxidase, BMI = body mass index, FBG = fasting blood glucose, FT3 = free T3, FT4 = free T4, HbA1c = hemoglobin A1c, HOMA-IR = HOMA insulin resistance score, TSH = thyroid stimulating hormone.

* In post hoc analysis, mild and moderate heterogeneity groups have statistically significant differences in weight.

† In post hoc analysis, mild and moderate heterogeneity groups have statistically significant differences regarding BMI.

‡ In post hoc analysis, moderate and high heterogeneity groups have statistically significant differences regarding spexin levels (*P* = .045).

**Table 3:** Comparison of the study’s variables according to the spexin levels when divided by tertiles.

Parameters	Tertile 1 n = 28	Tertile 2 n = 30	Tertile 3 n = 29	*P*-value
Gender (female)	23 (82.1)	22 (73.3)	24 (82.8)	.606
Co-morbidities, n (%)	0 (0.0)	0 (0.0)	0 (0.0)	–
Hashimoto disease, n (%)	14 (50.0)	11 (36.7)	20 (69.0)	**.045** *
Age (years)	31 (18–57)	33 (19–66)	38 (21–67)	.295
Height (cm)	165.82 ± 8.42	167.67 ± 8.07	162.79 ± 8.33	.080
Weight (kg)	71.46 ± 13.39	70.07 ± 12.85	68.69 ± 12.18	.717
BMI (kg/m^2^)	25.9 ± 3.91	24.74 ± 3.96	25.93 ± 4.39	.450
FBG (mg/dL)	83 (72–105)	86 (75–98)	88 (78–99)	.267
HbA1c (%)	5.5 (4.8–6.2)	5.3 (4.7–6)	5.4 (4.9–6.1)	.428
Insulin (mU/L)	8.18 ± 2.87	8.35 ± 2.65	7.89 ± 2.81	.815
fT3 (ng/L)	3.02 ± 0.39	3.22 ± 0.4	3.1 ± 0.43	.175
fT4 (ng/L)	12 (9.19–18)	12.1 (9–17.9)	13.3 (9.4–17)	.533
TSH (mU/L)	1.98 (0.12–12)	1.89 (0.29–6.31)	1.67 (0.01–8.58)	.905
Anti-TG (U/mL)	117.5 (11.8–1089)	182 (32–1007)	153 (14.4–1089)	.488
Anti-TPO (U/mL)	161.5 (9–422)	130 (9–451)	249 (9–600)	.626
HOMA-IR	1.76 ± 0.65	1.81 ± 0.58	1.73 ± 0.64	.892
Spexin level (pg/mL)	42.33 (42.19–42.99)	45.77 (43.01–47.41)	54.64 (47.8–405.35)	**<.001**

The bold values in the table indicate statistical significance.

Anti-TG = anti-thyroglobulin, anti-TPO = anti-thyroid peroxidase, BMI = body mass index, FBG = fasting blood glucose, FT3 = free T3, FT4 = free T4, HbA1c = hemoglobin A1c, HOMA-IR = HOMA insulin resistance score, TSH = thyroid stimulating hormone.

* In post hoc analysis, the patients with tertile 3 have higher Hashimoto disease rate when compared to the patients with tertile 2 (chi-square and post hoc Bonferroni adjusted *z* tests were used).

## 4. Discussion

In our study, we investigated the role of SPX, which has been shown to exhibit positive and sometimes negative correlations with cytokines associated with immunological complex processes involving many advanced pro-inflammatory cascades in various tissues, in the immunoetiopathogenesis of HT. We observed that high SPX and high antibody levels were correlated in HT patients. Increased endogenous SPX levels in the group with high antibody titers may have increased in order to protect, regulate, or suppress the immune response.

HT is a chronic and destructive disease of the thyroid that results in lymphocyte infiltration and fibrosis triggered by widely known environmental and immune factors, leading to clinical hypothyroidism.^[9]^

In our study, 87.7% of the patients in the HT group were female, consistent with the literature, and the mean age was significantly higher than in the control group. There are studies suggesting that the prevalence of HT increases with age, especially in patients with other autoimmune diseases such as myasthenia graves, systemic sclerosis, celiac disease, and Sjögren’s disease.^[11]^

HT has long been listed as one of the most common endocrinologic causes of obesity. In fact, weight gain is often the first sign of hypothyroidism. Even if TSH levels are normalized with levothyroxine replacement therapy, 82% of patients, especially women, continue to gain weight, and 35% struggle with obesity.^[12]^

In a meta-analysis of 22 studies by Song et al, there was a highly statistically significant correlation between obesity and HT patients, even though euthyroidism was achieved with levothyroxine replacement therapy. It was also observed that even if these patients became euthyroid, the rate of achieving a normal BMI was considerably lower than in non-HT patients.^[13]^

Although both HT patients and the control group who participated in our study had a BMI < 30 kg/m^2^, the BMI was statistically significantly higher in the HT group compared to the control group, in accordance with the literature. Studies have emphasized that HT patients with higher anti-TPO levels are more prone to obesity. In addition, it is known that both HT and obesity are linked to low-level chronic inflammation, and some common immune cytokines such as TNF-α and IL-6 are increased.^[13]^ In our study, similar to the literature, we found that HT patients with high anti-TPO levels had higher BMI levels than both the control group and other HT patients with low antibody titers. However, it is very difficult to explain this excess weight with increased antibody levels and moderate chronic inflammation in HT patients. As we know, thyroxine (T4) must be converted to triiodothyronine (T3) by the enzyme Type 2 deiodinase in order to show biological activation at the cellular level.^[14]^

Increased T3 at tissue level increases thermogenesis and energy expenditure in brown adipose tissue (BAT) by regulating gene transcription factors.^[14]^ BAT has positive effects on thermogenesis and energy expenditure and plays an important role in the etiopathogenesis of obesity. In patients with HT, even if they become euthyroid with replacement therapy, energy expenditure and thermoregulation will not be sufficient in BAT. T4–T3 conversion, due to type 2 deiodinase enzyme mutations, is not sufficient, and T3 cannot increase at the tissue level as a result, which will lead to weight gain.^[15]^

In our study, we attribute the higher T4 level in the HT group compared to the control group to levothyroxine replacement. T3 levels were similar in all patients participating in the study. The type 2 deiodinase enzyme is affected by many diseases and medical treatments. In our study, there were no additional comorbid diseases and medical treatments that could interact in both the HT and control groups. However, it should be noted that the type 2 deiodinase enzyme may differ between individuals due to gene polymorphism, and intracellular T3 levels may vary in each patient. This difference causes clinical heterogeneity independently of laboratory levels.^[16]^

Thyroid ultrasonography is an important noninvasive tool that helps clinicians diagnose, evaluate, and treat HT.^[17]^ Studies have shown that echogenicity on ultrasonography is a reliable standard to confirm diagnosis or determine therapeutic efficacy.^[18]^ Sun et al graded HT cases according to USG findings in their study.^[19]^ In our study, we divided our patients into 3 groups according to ultrasound findings and found that most of the HT group had TSH levels that increased with increasing heterogeneity, according to ultrasound data.^[20]^

Since our study was performed in euthyroid HT patients receiving replacement therapy, we did not have the chance to evaluate the well-known correlation between ultrasound findings and TSH, as expected. It is known that parenchymal infiltration and fibroplastic proliferation increase over time in the course of HT and have a more degenerated and fibrotic appearance ultrasonographically.^[21]^ In our study, it was observed that our patients had severe heterogeneity at the G3 level. In this patient group, prolonged disease duration was associated with an increased levothyroxine dose.

Kolodziejski et al showed that SPX was effective in the regulation of immune response by decreasing TNF-α and IL-6 levels in liver and serum in rats.^[9]^ Metabolic syndrome (MS) induction is associated with increased BMI, increased blood pressure, blood glucose, insulin, uric acid, advanced glycation end products, insulin resistance, IL-6, and TNF-α, along with dyslipidemia, low serum spexin, peroxisome proliferator-activated receptor-gamma, and adenosine monophosphate-activated protein kinase (AMPK) levels. It has been emphasized that SPX reduces IL-1β and TNF-α levels by inhibiting inflammation together with the activation of PAR-ɣ and AMPK in type-2 DM and MS, and with this effect, it can be used in the treatment.^[22,23]^

In many studies, it has been observed that in patients with HT, IL-17, IL-6, IL-21, IL-22, and IL-23 secretion, especially IL-17, is increased and expressed in excessive amounts in blood and thyroid tissue, which triggers autoimmune responses.^[24]^ Moreover, it has been suggested that several advanced pro-inflammatory cascades are involved in the immunological etiopathogenesis of HT and that multiple inflammatory components (NLRP1, NLRP3, NLRC4, AIM2, ASC, and caspase-1) and cytokines associated with these components, such as IL-18, IL-1β, interferon-gamma, and TNF-α, cause cell apoptosis and fibrosis.^[25]^ The parameters obtained by complete blood count and their relationship with certain diseases have recently attracted the attention of researchers. Neutrophil/lymphocyte ratio (NLR) is considered an indicator of inflammation, and a high neutrophil count predicts ongoing inflammation, while a decreased lymphocyte count is considered an indicator of poor prognosis. The combination of these 2 measurements is generally accepted to predict the inflammatory state. NLR reflects the inflammatory burden (neutrophil count) and regulatory mechanisms (lymphocyte count) in inflammatory diseases.^[26]^ In addition, the uric acid and HDL cholesterol ratio (UA/HDL) has been shown to increase in inflammatory conditions, MS, and type-DM. Based on the relationship between the UA/HDL ratio and inflammation, it has also been evaluated in patients with HT, and it has been emphasized that the UA/HDL ratio is a reliable and useful marker for HT.^[27]^

Furthermore, C-reactive protein is a new inflammatory marker associated with various conditions, such as C-reactive protein to lymphocyte count ratio, and has been studied and found to be elevated in patients with thyroiditis.^[28]^.

Although inflammatory markers have been shown to be increased in patients with HT thyroiditis, many studies have shown that there is no positive correlation between antibody levels and disease severity in HT. Antibody titers are shown to be higher during active periods of the disease and decrease over the years.^[29]^ Consistent with the literature, we found that antibody titers were lower in the G3 group, which had a longer disease duration and needed higher doses of levothyroxine replacement compared to the other groups.

We have not found any study in the literature evaluating the relationship between SPX expression from follicle epithelial cells in thyroid tissue and thyroid dysfunction. We found that serum SPX levels were similar in the HT and control groups. However, SPX levels were found to be borderline statistically significantly higher among HT patients, especially in the G2 group where antibody titers were higher. The absence of metabolic comorbid diseases made the groups quite homogeneous. Therefore, we consider the increased SPX levels in the G2 group highly significant, and increased SPX levels suggest a role in the immunoetiopathogenesis of HT. In previous studies, it was reported that SPX was negatively correlated with hs-CRP, IL-6, and TNF-α levels and positively correlated with IL-1β.^[30,31]^

In the literature, it is shown that SPX exhibits positive and sometimes negative correlations with cytokines involved in immunological complex processes, in which many advanced pro-inflammatory cascades are involved in the etiopathogenesis of immunological processes occurring in different tissues. After categorizing patients according to spexin levels, it is shown that in tertile 3, higher spexin levels were measured compared to tertiles 1 and 2 (*P* < .001). And it is noted that 20 out of 29 patients in tertile 3 were diagnosed with HT (Table 3). Therefore, increased endogenous SPX levels in the group with high antibody titers may have been increased in order to protect the body, regulate, or suppress the immune response. Just as SPX protects against MS by inhibiting inflammation along with activation of peroxisome proliferator-activated receptors-gamma and AMPK in patients with MS.^[23]^

The limitations of our study include the fact that SPX levels were not compared with the mentioned cytokines, being single-centered, having a small number of patients, and being cross-sectional.

## 5. Conclusion

In our study, we evaluated SPX levels in HT patients, and we believe that this study is new to the literature. We found that HT patients expressed high SPX with high antibody levels. Further studies are needed to understand this new marker, which has a complex effect on the inflammation process, and the nature of these effects has not yet been clarified. We believe that multicenter studies with high case series, especially at the tissue level, are needed to fully explain the role of SPX in HT immunoetiopathogenesis. The center of interest should be understanding the immune-checkpoint pathways that lead to the emergence of drugs that are frequently used in the treatment of various diseases recently and then to be used in treatment.

## Author contributions

**Conceptualization:** Oguzhan Aksu.

**Data curation:** Oguzhan Aksu.

**Formal analysis:** Oguzhan Aksu, Muhammet Cemal Kizilarslanoglu.

**Funding acquisition:** Oguzhan Aksu, Selma Ozlem Celikdelen, Betul Cigdem Yortanli.

**Investigation:** Oguzhan Aksu, Ummugulsum Can, Muhammet Cemal Kizilarslanoglu.

**Methodology:** Oguzhan Aksu, Muhammet Cemal Kizilarslanoglu.

**Project administration:** Oguzhan Aksu.

**Resources:** Oguzhan Aksu.

**Software:** Oguzhan Aksu.

**Supervision:** Oguzhan Aksu.

**Validation:** Oguzhan Aksu.

**Visualization:** Oguzhan Aksu.

**Writing – original draft:** Oguzhan Aksu, Ayse Gunay.

**Writing – review & editing:** Oguzhan Aksu, Ayse Gunay.
